# Trends in Pollination Scientists' Research: A Comprehensive Analysis in Citations and Research Topics

**DOI:** 10.1002/ece3.71215

**Published:** 2025-05-07

**Authors:** Ehsan Rahimi, Chuleui Jung

**Affiliations:** ^1^ Agricultural Science and Technology Institute Andong National University Andong Republic of Korea; ^2^ Department of Plant Medical Andong National University Andong Korea

**Keywords:** citation analysis, network analysis, pollination ecology, pollinators, research topics, trend analysis

## Abstract

In pollination ecology research, there is a notable lack of understanding regarding the evolving trends and changes in scientific topics, which hampers the field's ability to address crucial ecological questions. Closing these knowledge gaps is essential for pollination ecologists to protect pollinator populations and their critical ecological roles. To analyze the work of scientists in pollination ecology, we identified researchers through Google Scholar, focusing on those who listed “pollination” or “pollinators” in their profiles. We also analyzed the 40 most‐cited papers in pollination ecology by these scientists, each with over 1000 citations, using statistical tests to explore the relationships between citation counts and various influencing factors. We also examined the top 50 most frequently used bi‐grams in article titles by these scientists to identify trends in research topics. We identified 223 pollination ecology scientists who collectively authored 14,661 papers, accumulating 1,570,139 citations. On average, each scientist received 7040 citations, with a mean H‐index of 32.5. Notably, 67.8% of these citations came from papers where the scientists were not the first author. Analyzing the 40 most‐cited papers revealed no significant correlations between citation counts and potential influencing factors. However, 24 of these papers emphasized the global importance of pollinators and their declines. Our trend analysis showed an increase in publications starting around 1974, peaking in 2020, and then declining. Citations have decreased since 2010, likely indicating a shift towards more specialized research topics. The analysis highlights a continued focus on bee research, particularly honeybees ( *Apis mellifera* ), within pollination ecology. Despite this focus, there has been a decline in publications since 2020 and in citations since 2010, suggesting the need for diversifying research themes to maintain the field's impact and relevance.

## Graphical abstract

Pollination ecology has garnered increasing attention due to its fundamental role in biodiversity conservation and ecosystem services. However, a lack of understanding regarding evolving trends and research shifts poses challenges for addressing emerging ecological questions. Our analysis encompasses the contributions of 223 prominent scientists in the field, their collective 14,661 publications, and over 1.5 million citations. By evaluating citation patterns and trends in research topics, we uncover key insights, including a declining trend in publications since 2020 and citations since 2010, alongside a sustained emphasis on bees, particularly honeybees ( *Apis mellifera* ).

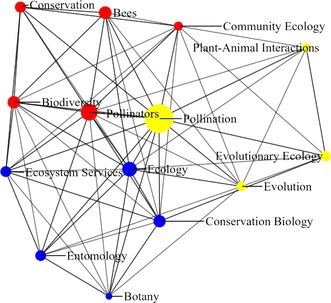

## 1 Introduction

Since Sprengel's seminal publication in 1793 (Sprengel and Sprengel 1793) on the interactions between flowers and insects that transport pollen to plant stigmas, demonstrating their crucial role in seed and fruit production, our understanding of pollination as an essential ecosystem process has significantly expanded (Mayer et al. 2011). Most flowering plant species in nearly all angiosperm‐dominated communities interact with pollinators, with an average interaction rate of 78% in temperate communities and 94% in tropical communities. This amounts to over 308,000 species, representing more than 87% of all angiosperms (Ollerton et al. 2011). Pollination by insects, or entomophily, originated in angiosperms approximately 120 million years ago (Schatz et al. 2017). Without pollinators, one‐third of flowering plant species would fail to produce seeds, while half would experience an 80% or greater decline in fertility (Rodger et al. 2021). The relationship between pollinators and flowering plants is mutualistic, playing a crucial role in the survival and stability of both communities (Liu et al. 2021).

Using FAO data for the years 1961–2006, Aizen et al. (2009) identified 87 pollinator‐dependent crops and estimated that without the presence of pollinators, between 3% and 8% of the world's total crop production would decrease. Production and consumption of pollinator‐dependent crops increased around the world between 1961 and 2006, especially in developed countries (16.7% vs. 9.4% increase in developing countries) (Aizen and Harder 2009). Global pollination benefits also had an increasing trend between 1993 and 2009 (Lautenbach et al. 2012). The economic importance of animal pollination in global agriculture varies significantly, estimated to range from $195 billion to $387 billion (Porto et al. 2020). Pollinators also play a crucial role in providing up to 40% of essential nutrients in the human diet (Eilers et al. 2011), with insect pollination alone contributing 9.5% to the total economic value of agricultural products directly consumed by humans (Gallai et al. 2009).

Developed nations such as Britain, Germany, and Japan are anticipated to face substantial economic impacts from pollinator declines (Murphy et al. 2022). This vulnerability underscores the potential shifts in plant–pollinator dynamics. In Brazil, where pollinator‐dependent crops accounted for 47% of dietary nutrients in 2017, declines in pollinator populations could result in nutritional losses ranging from 8% to 30% (Porto et al. 2021). Pollinating insects include moths, butterflies, bumblebees, honeybees, solitary bees, and hoverflies, beetles, and flies. Bees are the most important pollinator taxa, as 9.5% of the world's crop production comes from products that are pollinated by wild bees (Gallai et al. 2009). Honeybees and bumble bees visit more than 90% of the world's food crops (Doyle et al. 2020). Hoverflies are among the most important pollinators in the world (Miličić et al. 2018; Stanley et al. 2013), visiting at least 72% of the world's food crops, which are worth 300 billion dollars per year (Doyle et al. 2020; Rader et al. 2020). Butterflies and moths also visit 54% of the world's food products (Rader et al. 2020).

Pollination ecology has transitioned from focusing mainly on the natural history of plant–pollinator interactions to a discipline that integrates concepts and theories from plant population biology and evolutionary ecology (Knight et al. 2018). Currently, the world is grappling with a profound decline in biodiversity, exacerbated by extensive habitat destruction, degradation, and climate change driven by human activities. These factors significantly disrupt and impact plant–pollinator relationships worldwide (Cameron et al. 2011; Goulson et al. 2015; Potts et al. 2010; Rundlöf et al. 2015). In the realm of pollination ecology studies, there exists a notable gap in understanding how research trends have evolved over time and whether there have been shifts in the topics and methodologies embraced by scientists. This gap hinders a comprehensive grasp of how the field has progressed in addressing critical ecological questions, such as the impacts of habitat loss, climate change, and agricultural practices on pollinator populations and their interactions with plants. Addressing these gaps is essential for advancing our understanding of ecological dynamics and informing effective conservation strategies.

In this context, a quantitative trend analysis of the pollination ecology literature allows us to objectively examine changes in this research area over recent years (Knight et al. 2018). Trend studies are crucial for advancing the understanding of ecology, environmental dynamics, natural resource management, and biodiversity conservation (Lindenmayer et al. 2012). Such research provides a comprehensive view of the pollination ecology field, which is essential for understanding current research trends and identifying gaps (Borthakur and Singh 2018; Hussain 2017; Maris et al. 2018). For example, analyzing the papers of pollination scientists from their first publication to the present, based on citation counts and other metrics, is vital for uncovering significant gaps and potential outcomes. One key outcome is identifying seminal papers and scientists who have significantly contributed to the field. This helps establish a historical context and acknowledges the foundational work that has shaped current understanding and practice in pollination ecology. By examining the trajectory of scientific publications, citation patterns, and topic selection from the inception of the pollination ecology field to the present, the current study aims to address these gaps by conducting a comprehensive temporal analysis of pollination ecology research.

## 2 Methods

### 2.1 Scholars' Profiles

To survey and analyze scientists in the field of pollination ecology, our first task was to identify these scientists or at least the majority of those working in this field. We used Google Scholar as our primary resource for this purpose. Google Scholar not only provides comprehensive information about researchers, including their article lists, total citation counts, citations per article, H‐index, and i10‐index, but also the publication dates of their works. Additionally, it allows researchers to list up to five keywords on their pages to indicate their areas of activity or interest. By focusing on the keywords “pollination” and “pollinators,” we were able to identify all individuals with Google Scholar profiles who had used either of these terms in English on their pages. Subsequently, we recorded the Google Scholar profiles (URL) of each identified scientist.

### 2.2 Scholars' Full Information

The R package of “Scholar” (CRAN: Package scholar (r‐project.org)) can extract full information about each scientist from Google Scholar. We utilized this package to gather detailed information on each scientist, including their total citation count, H‐index, i10‐index, and a list of all their articles, along with the publication years, citation numbers, and full author lists for each article. Then, we extracted up to five keywords from their pages, which the scientists added to represent their preferred scientific topics. To address the challenges associated with web scraping Google Scholar data using the “scholar” package, we implemented a 5‐s delay between requests in our R code. This approach helped prevent access restrictions and avoided triggering Google Scholar's automated blocking mechanisms. Additionally, we monitored request responses to detect and handle potential errors, ensuring the extraction process remained stable.

### 2.3 Descriptive Statistics of Scholars' Citations

For all the scientists whose citation information we extracted, we calculated data such as the mean number of total citations, H‐index, and i10‐index. To determine the percentage of total citations each scientist earned through collaboration, we focused on citations from articles where the scientist was the first author. This allowed us to ascertain what proportion of their citations came from articles where they were not the first author. We focused on first‐author publications because, in many cases, the first author is primarily responsible for conducting the research and writing the manuscript. While last‐author positions often indicate a senior researcher or principal investigator directing the study, this is not universally consistent across disciplines or collaborations. Since Google Scholar does not explicitly categorize author roles beyond listing names in order, our approach ensured a more standardized and reproducible method for estimating collaboration contributions. However, we acknowledge that incorporating last‐author positions could provide additional insights into research leadership, and future studies with more detailed authorship metadata could explore this aspect further.

### 2.4 Keyword Network Visualization

In this section, we aimed to determine whether scientists in the field of pollination ecology could be categorized into distinct clusters based on the keywords listed on their profiles. We utilized keyword network visualization to offer a comprehensive overview of the interconnected themes present in the publication titles of these scholars. This analysis not only highlights their primary focus on pollination but also reveals the additional fields of interest within this scientific community. By examining the keywords that frequently co‐occur with “pollination” or “pollinators,” we identified patterns and connections that suggest broader research interests among these scientists.

### 2.5 A Review of the Most‐Cited Papers in the Field of Pollination Ecology

To identify the most influential studies in pollination ecology, we analyzed the citation records of the scientists in our study and selected 40 papers that had each accumulated at least 1000 citations. This threshold was chosen to ensure that we focused on publications with significant academic impact while maintaining a feasible dataset size. If we had chosen a lower threshold, such as 500 citations, the number of papers to assess would have expanded significantly, potentially reaching hundreds or thousands, making the analysis impractical. A higher citation threshold also allowed us to highlight studies that have demonstrated long‐term relevance and influence within the scientific community.

We did not include the most recently published papers because they have not yet had sufficient time to accumulate citations, which is a time‐dependent process. Relying on such papers would introduce bias, as their citation counts may not accurately reflect their actual long‐term impact. Including only highly cited papers ensures that we capture research that has already undergone a sustained period of academic recognition, reducing the risk of premature assessments based on fluctuating citation patterns. Given that recent studies may still be in the early stages of citation accumulation, they might not yet have had the opportunity to shape the field to the same extent as older, highly cited works.

For each of these articles, we recorded the title, number of authors, journal, and citation count, and categorized them based on their focus. The categories included: (1) the importance of pollinators, which emphasizes the role of pollinators in flower pollination and food security; (2) pollinator declines, highlighting factors such as insecticides and climate change that threaten pollinator populations; (3) landscape modification, discussing the effects of landscape structure on pollinators and strategies like planting flower patches to attract them; (4) pollination networks, focusing on the interactions and structural features of plant–pollinator networks; (5) pollinator behavior, analyzing foraging behaviors with theories like central place forager theory; and (6) the ecology of pollinators, which explores the ecology and natural history of pollinators. Additionally, we determined the main outcome or goal of each article and noted whether they were published in open‐access or subscription‐based journals.

### 2.6 Statistical Tests Citations Analysis

Understanding why these studies received the most citations was crucial. Specifically, we wanted to determine if factors such as the number of authors, publication year, article category, or open access status had a statistically significant relationship with citation counts. To explore this, we employed Pearson's correlation test to examine the relationship between the number of authors and the year of publication. For assessing the impact of open access on citation counts, we used the Wilcoxon rank sum test (also known as the Mann–Whitney U test), a nonparametric test. The hypotheses for this test were:

Null Hypothesis H0 *There is no difference in citation counts between open‐access and nonopen‐access papers*.

Alternative Hypothesis H1 *There is a difference in citation counts between open‐access and nonopen‐access papers*.

Additionally, to determine if the assigned category affected citation rates, we applied the Kruskal–Wallis test. The hypotheses for this test were:

Null Hypothesis H0 *There is no difference in median citation counts across the categories*.

Alternative Hypothesis H1 *At least one category has a different median citation count compared with the others*.

### 2.7 Co‐Authorship Network Visualization

We aimed to identify which scientists had the most collaboration in writing the 40 most‐cited articles and to determine if we could group the authors into distinct clusters based on their collaborations. In other words, we sought to find out whether there is a core group of scientists within the field of pollination ecology to which these articles predominantly belong. To achieve this, we conducted a network analysis of the authors, focusing on their co‐authorships, to reveal which scientists had the highest levels of cooperation with each other.

### 2.8 Trends Analysis for all Papers and Citations

To assess long‐term trends in scientific contributions, we analyzed the complete list of articles and corresponding citation counts for each scientist included in our study. This approach enabled us to track annual trends in both publication output and total citations, revealing shifts in research activity and influence over time. Importantly, we did not limit our analysis to papers specifically related to pollination ecology; instead, we considered all articles listed in each scientist's Google Scholar profile to provide a comprehensive view of their academic trajectory. This method ensures consistency across researchers, as their publication portfolios may span multiple disciplines, including but not limited to pollination ecology. Our goal was to identify any patterns or shifts over time in the publication and citation rates of these scientists. This trend analysis provides insights into the evolution and development of the field of pollination ecology across different years.

To understand which keywords appear most frequently in the titles of articles by scientists in the field of pollination ecology and how many citations these articles have received, we identified the top five most frequently used words along with their citation counts. This initial analysis aims to uncover any trends in the topics of interest among scientists over time. However, since this approach relies solely on individual word frequency and may not fully capture trends in the field of pollination ecology, we conducted a more sophisticated analysis. This involved examining pairs of words that appear sequentially in article titles, known as bi‐grams. We identified up to 50 such bi‐gram combinations and analyzed their frequency of occurrence and corresponding citation counts. This comprehensive approach provides deeper insights into evolving research interests and trends within the field of pollination ecology.

## 3 Results

### 3.1 Summary of Scholar Profiles

In total, using Google Scholar data, we identified 223 scientists actively working in the field of pollination ecology. The combined total number of citations for these 223 scientists is 1,570,139. Table 1 presents the descriptive statistics of various citation metrics for scientists. On average, these scientists have accumulated a total of 7040 citations throughout their careers, with a mean H‐index of 32.5, indicating a high level of impact in their field. The i10‐index, which measures the number of publications with at least 10 citations each, averages 62, highlighting the breadth of their scholarly contributions. When considering articles where they are the first author, these scientists have received a total average of 1984. Moreover, the mean percentage of citations derived from collaborative works stands at 67.8%, indicating that a significant portion of their citations is generated through collaborations with other researchers (see Data S1 for more details).

**TABLE 1:** Mean of total citations, H‐index, i10 index, mean total citations for authors as first authors, and mean percentage of citations based on collaboration.

	Total citation	H‐Index	i10‐index	First author	Collaboration proportion %
Mean	7040	32.5	62	1984	67.8
SD	11,159	19.1	54.2	2851	18.5

### 3.2 Keyword Network Visualization

**FIGURE 1:**
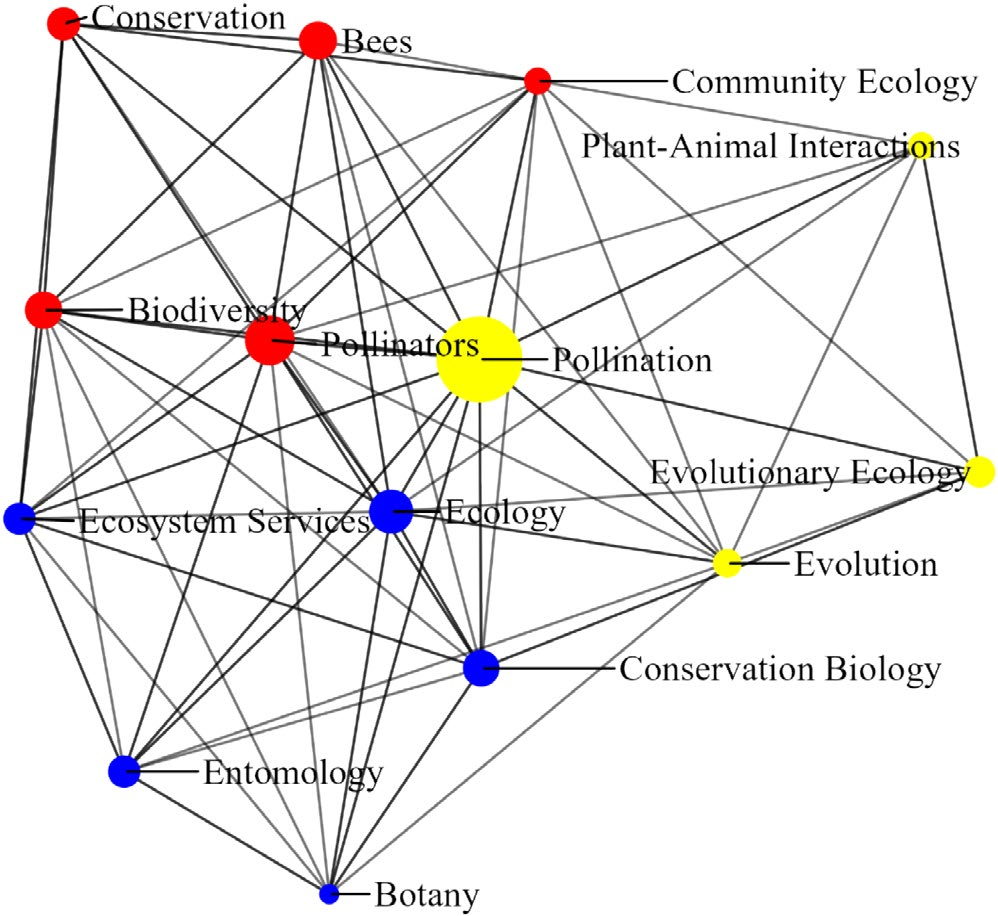
Network visualization of Google Scholar keywords with a minimum frequency of 10.

We have gathered comprehensive data from 223 scientists, each of whom has provided a total of 982 distinct keywords to denote their areas of interest and research topics. Among these keywords, the most frequently mentioned ones include “pollination” with 182 mentions, followed by “pollinators” with 45, “ecology” with 33, “bees” with 23, and “biodiversity” with 22. Figure 1 illustrates the network of keywords with a minimum frequency of 10, revealing three distinct clusters or components represented by different colors. Community 1 includes community ecology, bees, pollinators, conservation, and biodiversity keywords. This community signifies research focused on the interactions within ecological communities, particularly regarding pollinators such as bees. Keywords like “community ecology” and “pollinators” emphasize understanding the roles and dynamics of various species within ecosystems, particularly those crucial for pollination.

Community 2 includes ecology, ecosystem services, botany, entomology, and conservation biology keywords. This community integrates broad ecological principles with applied conservation practices. This community represents interdisciplinary research aimed at understanding and preserving natural ecosystem functions for the benefit of biodiversity and society. Community 3 includes pollination, evolution, plant–animal interactions, and evolutionary ecology keywords. The community centers on the evolutionary aspects of ecological interactions, particularly in pollination. This community reflects research exploring how evolutionary dynamics influence ecological relationships and drive evolutionary processes. The identified communities within the keyword network provide a comprehensive view of the major research themes in pollination ecology and related fields. Together, these communities highlight the diverse and interconnected nature of research in this field, addressing both fundamental scientific inquiries and applied conservation challenges (see Data S1 for more details).

Table 2 summarizes the dataset details of the 40 most‐cited papers in the field of pollination ecology. Each entry in the dataset includes the reference, title, number of authors, journal, citation count, category, main outcome, and accessibility status (open access or not). On average, these papers are authored by approximately 7.95 authors, with the number of authors per paper ranging from 1 to 52. The citation count for these papers varies widely, spanning from 1003 to 8329 citations and averaging around 2481 citations per paper. The papers are categorized into several key areas: Importance of pollinators (10 papers), pollinator declines (14 papers), landscape modification (7 papers), pollinator behavior (5 papers), pollination networks (4 papers), and pollinator ecology (1 paper) (see Data S1 for more details).

**TABLE 2:** Title, number of authors, journal, number of citations, category, main outcome, and accessibility of 40 most‐cited papers in the field of pollination ecology.

Reference	Title	Authors	Journal	Citation	Category	Outcome	Open‐access
Klein et al. (2007)	Importance of pollinators in changing landscapes for world crops	7	Proceedings of the Royal Society B: Biological Sciences	8329	Importance of pollinators	87 of the leading global food crops are dependent upon animal pollination	Yes
Potts et al. (2010)	Global pollinator declines: trends, impacts and drivers	6	Trends in Ecology & Evolution	7087	Pollinator declines	Global review of the potential drivers of pollinator loss	Yes
Tscharntke et al. (2005)	Landscape Perspectives on agricultural intensification and biodiversity–ecosystem service management	5	Ecology Letters	5270	Landscape modification	Agricultural land use needs a landscape perspective to effectively conserve biodiversity and ecosystem services	No
Pyke et al. (1977)	Optimal foraging: a selective review of theory and tests	3	The quarterly Review of Biology	4287	Pollinator behavior	Optimal foraging theory is supported by data but needs refinement to address complex situations and currencies beyond energy	No
Ollerton et al. (2011)	How many flowering plants are pollinated by animals?	3	Oikos	4147	Importance of pollinators	About 88% of flowering plants are pollinated by animals	Yes
Biesmeijer et al. (2006)	Parallel declines in pollinators and insect‐pollinated plants in Britain and the Netherlands	12	Science	3988	Pollinator declines	Pollinator declines were most common among habitat and flower specialists, univoltine species, and nonmigrants	No
Goulson et al. (2015)	Bee declines are driven by combined stress from parasites, pesticides, and lack of flowers	4	Science	3897	Pollinator declines	Species richness of wild bees and other pollinators has declined significantly over the past 50 years	No
Goulson et al. (2015)	Wild pollinators enhance fruit sets of crops regardless of honeybee abundance	50	Science	2872	Importance of pollinators	Wild pollinators enhance crop fruit sets regardless of the number of honeybees present	No
Pyke (1984)	Optimal foraging theory: a critical review	1	Annual Review of Ecology, Evolution, and Systematics	2802	Pollinator behavior	Acritical review of optimal foraging theory	Yes
Roubik and Roubik (1992)	Ecology and natural history of tropical bees	2	Cambridge University Press	2640	Pollinator ecology	Ecology and Natural History of Tropical Bees	No
Fenster et al. (2004)	Pollination syndromes and floral specialization	5	Annual Review of Ecology, Evolution, and Systematics	2522	Pollination networks	Pollination syndromes are valuable for understanding floral diversification mechanisms by organizing pollinators into functional groups	Yes
Cox‐Foster et al. (2007)	A metagenomics survey of microbes in Honeybee Colony Collapse disorder	22	Science	2404	Pollinator declines	One organism, the Israeli acute paralysis virus of bees, was strongly correlated with colony collapse disorder	No
Waser et al. (1996)	Generalization in pollination systems, and why it matters	5	Ecology	2285	Pollination networks	Pollination systems often exhibit more generalization and dynamics than traditional views of specialization suggest	No
Cameron et al. (2011)	Patterns of widespread decline in North American bumble bees	7	Proceedings of the National Academy of Sciences	1963	Pollinator declines	Declines in North American bumble bee populations, linked to higher Nosema bombi infection rates and reduced genetic diversity	Yes
Greenleaf et al. (2007)	Bee foraging ranges and their relationship to body size	4	Oecologia	1906	Pollinator behavior	Body size and sociality distinctly influence both the potential and actual foraging movements of bees.	Yes
Kremen et al. (2007)	Pollination and other ecosystem services produced by mobile organisms: a conceptual framework for the effects of land‐use change	19	Ecology Letters	1865	Landscape modification	Managing ecosystem services provided by mobile organisms, such as pollination, requires understanding their foraging range	Yes
Henry et al. (2012)	A common pesticide decreases foraging success and survival in honeybees	9	Science	1850	Pollinator declines	Nonlethal thiamethoxam exposure disrupts honeybee homing, jeopardizing colony survival and highlighting global pesticide risks	No
Potts et al. (2016)	Safeguarding pollinators and their values to human well‐being	11	Nature	1791	Importance of pollinators	Pollinators provide vital benefits to society but face multiple threats	No
VanEngelsdorp et al. (2009)	Colony collapse disorder: a descriptive study	13	PloS one	1698	Pollinator declines	A descriptive study of colony collapse disorder	Yes
Whitehorn et al. (2012)	Neonicotinoid pesticide reduces bumble bee colony growth and queen production	4	Science	1630	Pollinator declines	Neonicotinoid pesticide decreases bumble bee colony growth and the production of queens	No
Ricketts et al. (2008)	Landscape effects on crop pollination services: are there general patterns?	13	Ecology Letters	1608	Landscape modification	Exponential declines in pollinator richness and native visitation rate with proximity to natural habitats	Yes
Vanbergen and Insect Pollinators Initiative (2013)	Threats to an ecosystem service: pressures on pollinators	1	Frontiers in Ecology and the Environment	1594	Pollinator declines	Mathematical modeling and simulations reveal that increased mortality and reduced carrying capacity of pollinators	Yes
Buchmann and Nabhan (2012)	The forgotten pollinators	2	Island Press	1577	Importance of pollinators	The vital role of pollination, emphasizing its contribution to one‐third of the world's food supply	No
Steffan‐Dewenter et al. (2002)	Scale‐dependent effects of landscape context on three pollinator guilds	5	Ecology	1546	Landscape modification	The abundance and diversity of solitary wild bees correlated positively with seminatural habitats at small scales up to 750 m	No
Delaplane and Mayer (2000)	Crop pollination by bees	2	CABI Publishing	1451	Importance of pollinators	Addressing the biology of pollination, culturing, and managing bees for optimum crop pollination	No
Aizen and Harder (2009)	The global stock of domesticated honeybees is growing slower than the agricultural demand for pollination	2	Current Biology	1446	Importance of pollinators	Managed honeybee presence reduces pollination limitation in crops that can self‐pollinate, but not in those requiring cross‐pollination	Yes
Hegland et al. (2009)	How does climate warming affect plant–pollinator interactions?	5	Ecology Letters	1418	Pollination networks	Climate warming impacts the phenology and distribution of plants and pollinators	Yes
Kennedy et al. (2013)	A global quantitative synthesis of local and landscape effects on wild bee pollinators in agroecosystems	41	Ecology Letters	1349	Landscape modification	Wild bee abundance and richness are positively influenced by diversified and organic farm management practices	Yes
Allen‐Wardell et al. (1998)	The potential consequences of pollinator declines on the conservation of biodiversity and stability of food crop yields	22	Conservation Biology	1343	Pollinator declines	The potential consequences of pollinator decline on biodiversity conservation and food crop yield stability	Yes
Gathmann and Tscharntke (2002)	Foraging ranges of solitary bees	2	Journal of Animal Ecology	1304	Pollinator behavior	Solitary bees have maximum foraging distances of 150–600 m	Yes
Winfree et al. (2009)	A meta‐analysis of Bees' responses to anthropogenic disturbance	5	Ecology	1265	Pollinator declines	Bee abundance and species richness are significantly, and negatively affected by disturbance	Yes
Kevan and Baker (1983)	Insects as flower visitors and pollinators	2	Annual Review of Entomology	1254	Pollination networks	Describing Insects as flower visitors and pollinators	Yes
Burkle et al. (2013)	Plant–pollinator interactions over 120 years: loss of species, co‐occurrence, and function	3	Science	1252	Pollination networks	The decline in the structure and function of interaction networks and the local extinction of half of bee species.	No
Rundlöf et al. (2015)	Seed coating with a neonicotinoid insecticide negatively affects wild bees	11	Nature	1193	Pollinator declines	Neonicotinoid seed coatings reduce wild bee density, solitary bee nesting, and bumblebee colony growth and reproduction, with no apparent effect on honeybees	No
Garibaldi et al. (2011)	Stability of pollination services decreases with isolation from natural areas despite honeybee visits	23	Ecology Letters	1121	Landscape modification	At 1 km from adjacent natural areas, spatial stability of pollination services decreased by 25%, 16%, and 9% for richness, visitation, and fruit set.	Yes
Buchmann (1983)	Buzz pollination in angiosperms	1	CABI publishing	1054	Pollinator behavior	The characteristics of vibrational pollination in angiosperms are summarized and reviewed	No
Klein et al. (2003)	Fruit set of highland coffee increases with the diversity of pollinating bees	3	Proceedings of the Royal Society of London. Series B: Biological Sciences	1040	Importance of pollinators	The fruit yield of coffee was correlated with the diversity of bee species visiting flowers, ranging from approximately 60% (with three species) to 90% (with 20 species)	Yes
Rader et al. (2016)	Nonbee insects are important contributors to global crop pollination	52	Proceedings of the National Academy of Sciences	1025	Importance of pollinators	Nonbees performed 25%–50% of the total number of flower visits	Yes
Tylianakis et al. (2007)	Habitat modification alters the structure of tropical host–parasitoid food webs	3	Nature	1017	Pollinator declines	Habitat modification significantly alters the structure of food webs for cavity‐nesting bees, wasps, and parasitoids in tropical regions	Yes
Higes et al. (2008)	How natural infection by Nosema ceranae causes honeybee colony collapse	11	Environmental Microbiology	1003	Pollinator declines	Nosema ceranae causes honeybee colony collapse	Yes

### 3.3 Statistical Analysis for Assessing Citations

Table 3 displays the pairwise Pearson correlation coefficients between three variables: citation count, number of authors, and year of publication for the 40 most‐cited papers in pollination ecology. The table reveals a weak negative correlation of −0.113 between citation count and number of authors, which is not statistically significant (*p* = 0.486). This indicates there is no strong evidence of a meaningful relationship between these variables in this sample. Additionally, the correlation between the year of publication and citation count is −0.048, also a very weak negative correlation. This suggests that the age of the papers does not significantly influence their citation count, but the relationship is negligible and not statistically significant (*p* = 0.767). Overall, these findings imply that for these 40 papers, the number of citations is not strongly linked to either the number of authors or the year of publication.

**TABLE 3:** Pearson correlation test results for the relationship between the number of citations, authors, and publication year.

Sample 1	Sample 2	*N*	Correlation	95% CI for *ρ*	*p*
Citation	Number of authors	40	−0.113	(−0.410, 0.205)	0.486
Citation	Publication year	40	−0.048	(−0.355, 0.267)	0.767

The results of the Wilcoxon rank sum test for assessing whether being an open‐access paper affects the number of citations showed the following: The test statistic (*W*) was calculated to be 251, with a *p* value of 0.1335. Since the *p* value is greater than the commonly used significance level of 0.05, there is no significant evidence to reject the null hypothesis. This means we fail to reject the null hypothesis that there is no difference in citation counts between open‐access and nonopen‐access papers. Therefore, the results suggest that, in this dataset, there is insufficient statistical evidence to conclude that open‐access status significantly influences citation counts. Overall, the Wilcoxon rank sum test indicates that open‐access status does not appear to meaningfully affect citation counts in the studied papers.

The results of the Kruskal–Wallis rank sum test for assessing whether the category assigned to each paper affects its number of citations showed the following: The Kruskal–Wallis chi‐squared value was 1.2217, with 5° of freedom, and a *p* value of 0.942. The *p* value of 0.942, which is significantly higher than the commonly used significance level of 0.05, indicates no statistically significant evidence to reject the null hypothesis. Therefore, the test results suggest that there is no significant difference in median citation counts across the different categories assigned to papers. In conclusion, the Kruskal–Wallis test indicates that the category to which a paper is assigned does not have a significant impact on its median citation counts, implying that category assignment does not influence citation performance based on this analysis.

### 3.4 Component Analysis of Co‐Authorship

**FIGURE 2:**
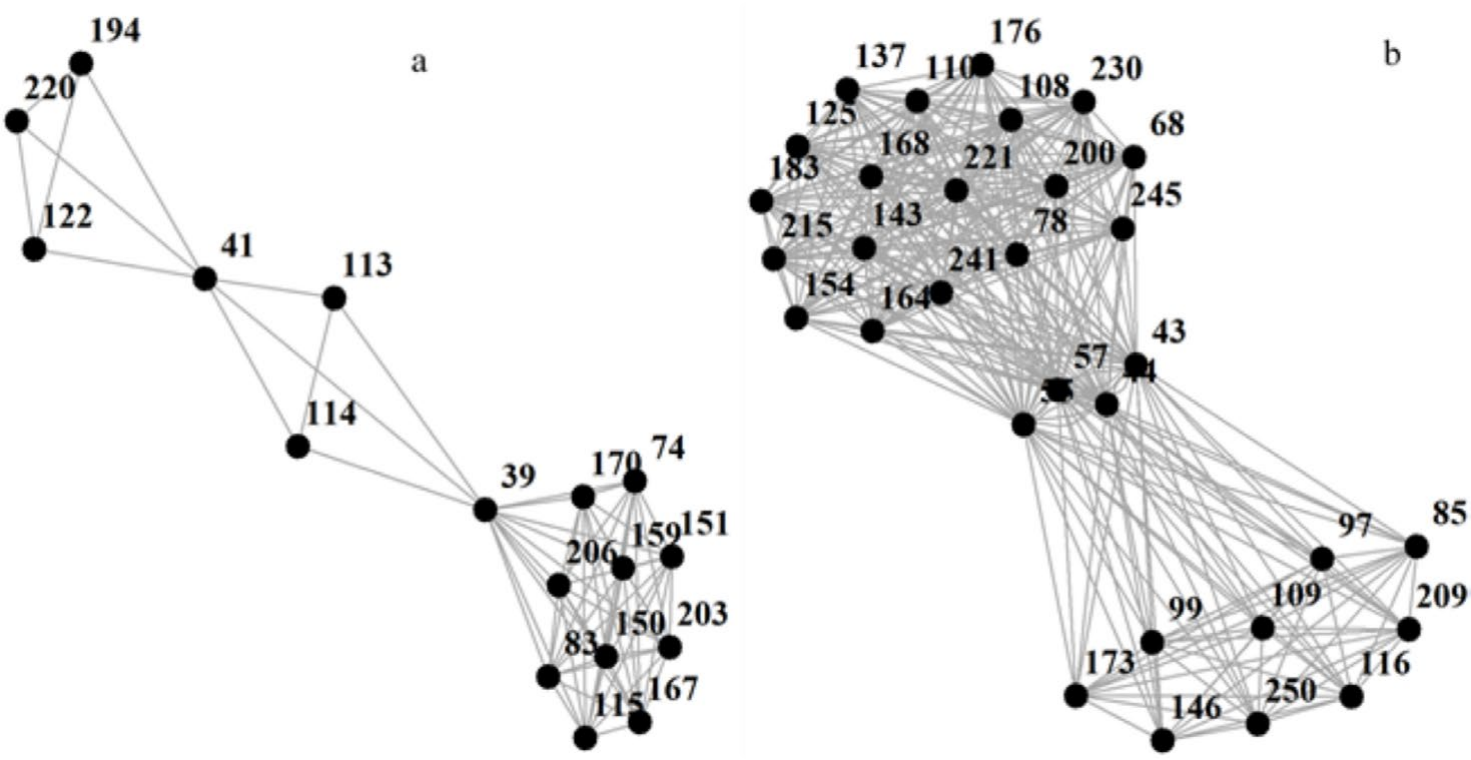
Two examples of the results of component analysis for co‐authorship of the most 40 cited papers: (a) Component 7 and (b) Component 12.

Overall, across the set of 40 papers, we identified a total of 250 distinct authors. The analysis of co‐authorship patterns among the 40 most‐cited papers in pollination ecology revealed a complex network characterized by 12 distinct components. Component 1 comprises 21 authors, forming the largest and most interconnected group within the dataset. Component 2 consists of 2 authors, representing a small, isolated collaboration. Component 3 is the second‐largest group, involving 77 authors who are extensively interconnected through co‐authorships. Component 4 includes two authors, indicating another small, isolated collaboration. Component 5 encompasses three authors, forming a small yet cohesive group of researchers. Component 6 consists of three authors, similarly representing a compact collaboration. Component 7 (Figure 2a) includes 17 authors, forming a substantial cluster indicative of a robust network of collaboration. Component 8 comprises five authors, reflecting a moderately interconnected group within the dataset. Component 9 contains five authors, indicating another moderately interconnected group. Component 10 involves 7 authors, while Component 11 comprises nine authors, both representing medium‐sized clusters of collaboration. Component 12 (Figure 2b) is notable for its size, involving 27 authors who are interconnected through co‐authorships, indicating a significant network of collaborative research efforts. These components collectively illustrate the diverse structures of collaborative networks among top‐cited authors in pollination ecology. The presence of multiple distinct components underscores the varied nature of research teams and collaborative patterns within the field (see Data S1 for more details).

### 3.5 Trends in the Number of all Papers and Citations

**FIGURE 3:**
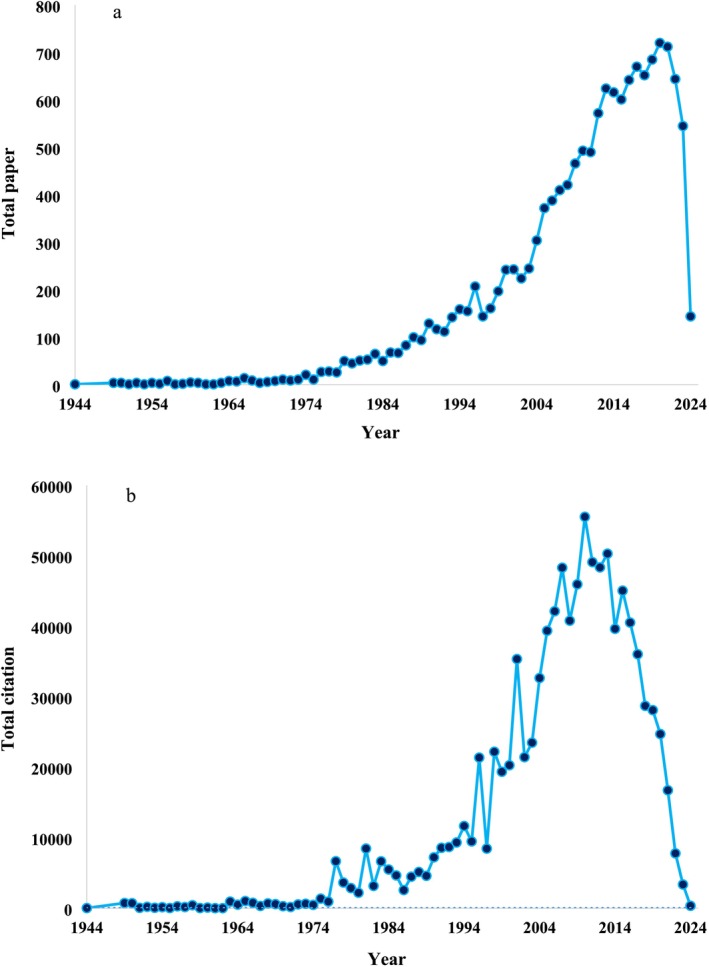
Trends in the total number of papers (a) and total citations (b) authored by 223 scientists in the field of pollination ecology.

In total, from 223 scientists, we extracted 14,661 papers after excluding papers without a publication year and those in other languages. The total citation count for all these papers was 1,022,797. Figure 3 illustrates the trends in the total number of papers (a) and total citations (b) authored by 223 scientists specializing in pollination ecology. The dataset, comprising approximately 14,600 articles, dates to 1944, with a notable increase in publications starting around 1974. By 2020, these scientists had published 720 articles, but since then, there has been a noticeable decline, continuing through July 10, 2024, when data collection for this study concluded, with 144 articles published during 2024 (Figure 3a). A similar pattern emerges for total citations, which saw a steady increase from 1974 onwards, peaking in 2010 with 55,527 citations. However, since 2010, there has been a decline in total citations, reaching 3360 by 2023 (Figure 3b).

**FIGURE 4:**
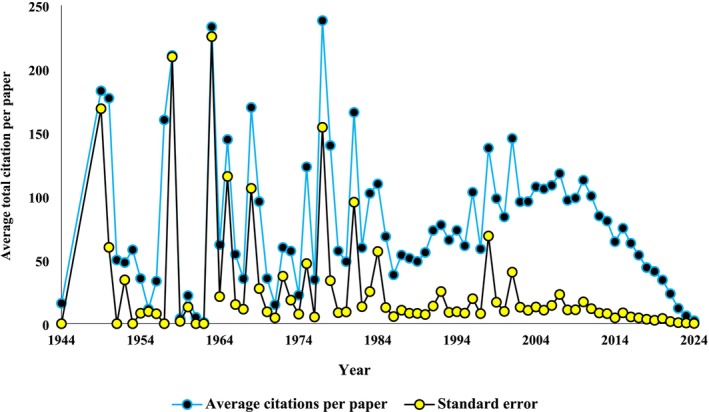
Trends in the average paper citation per year authored by 223 scientists in the field of pollination ecology.

Figure 4 depicts trends in the average citations per paper per year authored by 223 scientists specializing in pollination ecology. The figure reveals distinct trends in citation patterns over time. In the early years between 1944 and 1976, citation counts remained relatively low and fluctuated significantly. However, a gradual increase began in the mid‐1960s, with occasional spikes, such as in 1963 and 1975. A notable turning point occurred in 1977, where citations jumped to 6662, marking a significant shift in research impact. From 1977 to 1995, citation numbers continued to grow rapidly, reflecting an increasing scholarly focus on the field. By the early 1980s, citations exceeded 8000 per year. This upward trend continued into the 1990s, with some years, such as 1990 and 1994. A period of exponential growth occurred between 1996 and 2010, where citation numbers surged dramatically. Following 2011, a gradual decline in citations became apparent. While numbers remained relatively high initially, with 2011 recording 49,099 citations and 2013 reaching 50,297, a downward trend began to emerge after 2014, fields drawing attention away from the topics covered in these publications.

**FIGURE 5:**
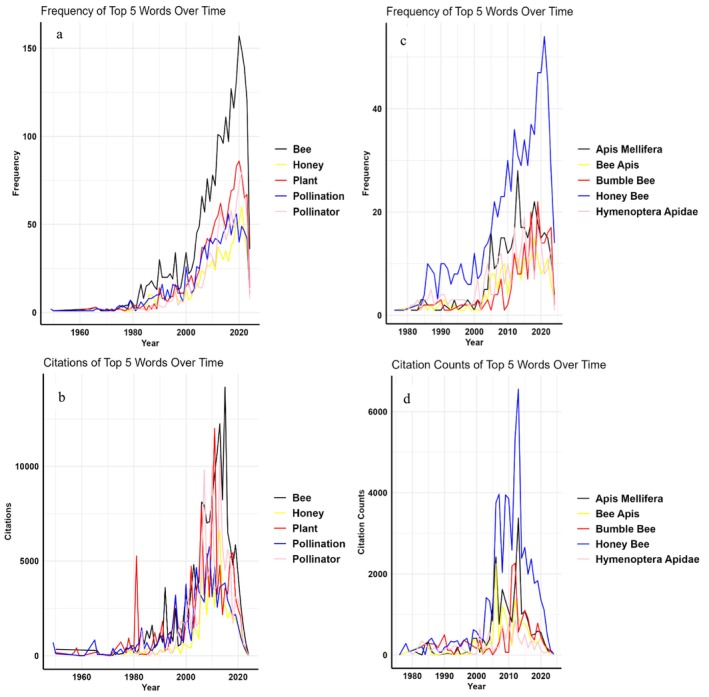
Trends in the total number of single keywords (a), related total citations (b), bi‐gram keywords (c), and (d) bi‐gram citations authored by 223 scientists in the field of pollination ecology.

Figure 5 presents detailed trends in keyword usage and citation patterns among 223 scientists specializing in pollination ecology. The figure is divided into panels showing the trends for single keywords (a) and their corresponding total citations (b), as well as for bi‐gram keywords (c) and bi‐gram citations (d). Firstly, the most frequently occurring single keywords in the titles of articles authored by these scientists include “bee,” “plant,” “pollinator,” “pollination,” and “honey.” The *Y*‐axis or frequency in these figures represents the total count of occurrences of the keywords in the titles of papers authored by researchers. These keywords not only dominate in terms of frequency but also in the number of citations they receive. The data indicate a peak in the occurrence of these single keywords around 2021, followed by a noticeable decline in subsequent years. However, when examining their citations, a similar trend of peaking around 2010 is observed, with a subsequent decline.

Notably, there are nuanced differences in the citation trends among these keywords post‐2010, suggesting varying impacts or research emphases over time. Moving to bi‐gram keywords, which are pairs of words frequently appearing together in article titles, prominent examples include “honey bee,” “ *Apis mellifera* ,” “bumble bee,” “Hymenoptera Apidae,” and “Apis bee.” However, the citation patterns for these bi‐grams display more variability. For instance, “ *Apis mellifera* ” shows a decline in citations starting around 2012, while others like “honeybee” and “bumble bee” started declining from around 2017. In summary, Figure 4 highlights the trends shift in research focus, changes in scientific interest, and varying levels of impact among different keywords and bi‐grams in the field of pollination ecology (see Data S1 for more details).

## 4 Discussion

### 4.1 Scholars' Profile Summary

From a total of 223 scientists in the field of pollination ecology, we found that these scientists have amassed 1,570,139 citations. The average scientist has received 7040 citations, with a mean H‐index of 32.5, indicating a substantial impact in their field. Additionally, 67.8% of their citations come from collaborative works, underscoring the importance of research partnerships. On the positive side, it indicates robust interdisciplinary research, leading to comprehensive and innovative findings. Collaboration often results in higher citation rates, broader impact, and better resource sharing, enhancing the quality of research. It also expands professional networks, offering more opportunities for future projects and career growth. However, there are potential drawbacks to this reliance on collaborative work. It may suggest lesser individual impact and can dilute personal recognition due to shared authorship. Managing large collaborative projects can also be challenging, requiring significant coordination and communication. Additionally, there may be institutional pressures to collaborate, potentially leading to partnerships that are more strategic than meaningful. Overall, while high collaborative citation rates are beneficial, balancing them with strong independent research is crucial for a dynamic and impactful scientific community. The keyword network visualization provides several insights into the research interests of the 223 scientists in the study. The frequent mention of keywords like “pollination,” “pollinators,” “ecology,” “bees,” and “biodiversity” in the researcher profiles confirms that these scientists are predominantly focused on pollination ecology, validating the selection of this group for trend analysis in this field.

### 4.2 Keyword Network Analysis and Co‐Authorship

The keyword network and co‐authorship analyses provided insights into how researchers in pollination ecology define their expertise and collaborate within the field. The keyword analysis, derived from Google Scholar profiles, reflects how scientists perceive and categorize their own research. The presence of distinct clusters suggests that researchers tend to align themselves with specific subfields, such as community ecology, evolutionary ecology, or conservation. This self‐assigned classification influences how pollination ecology is studied and communicated, as it shapes networking opportunities, funding priorities, and research collaborations. However, because these keywords are self‐reported rather than systematically assigned, they may also reflect individual preferences or trends in scientific branding rather than objective research categorization. This could lead to biases where certain emerging or interdisciplinary topics might be underrepresented simply because researchers do not explicitly label their work in those terms.

The co‐authorship analysis, on the other hand, highlights the structure of academic collaboration in pollination ecology. The existence of multiple co‐authorship clusters suggests that while some researchers work within highly interconnected networks, others collaborate more selectively or within smaller teams. The largest co‐authorship components indicate key research hubs, where influential scientists contribute to shaping the field through extensive collaboration. Meanwhile, the presence of smaller, isolated groups may reflect niche research areas, regional limitations, or institutional constraints on collaboration. Notably, because this analysis is based on the most‐cited papers, it also reflects the degree to which high‐impact research is produced within large networks versus independent or small‐team efforts. These findings suggest that while collaborative efforts dominate pollination ecology, there is still fragmentation in the field, which may affect knowledge exchange and the integration of diverse research perspectives. Encouraging greater cross‐network collaboration could help bridge gaps between specialized research clusters, fostering a more integrated and comprehensive approach to pollination ecology research.

### 4.3 The 40 Most‐Cited Papers

The analysis of the 40 most‐cited papers in pollination ecology revealed that citation counts were not significantly correlated with the year of publication or the number of authors. Pearson correlation analysis showed weak negative correlations that were not statistically significant, suggesting that these factors do not systematically influence citation frequency within this field. Additionally, the Wilcoxon rank sum test indicated that open‐access status does not significantly impact citation counts, while the Kruskal–Wallis rank sum test found no significant differences in citation counts among different paper categories.

Previous studies, such as those by Leimu and Koricheva (2005a, 2005b), have identified factors that influence citation rates in ecological research, including study direction, paper length, number of authors, and international collaborations. While these factors have been shown to affect citation rates in broader ecological literature, our findings suggest that their influence may be less pronounced in pollination ecology. One potential explanation is that pollination ecology research is often driven by conservation urgency and societal relevance, which may play a more significant role in determining citation frequency than structural elements like authorship patterns or institutional affiliation.

Although citation counts are often criticized as a proxy for research quality due to their susceptibility to biases (e.g., self‐citations, field size, or journal impact factors), they remain a widely used indicator of scientific influence. While we acknowledge the limitations of citation‐based assessments, the high citation rates of studies on pollinator declines and their ecological importance suggest that research with direct conservation applications tends to gain more visibility. This trend aligns with the growing concern over pollinator loss and its implications for biodiversity and food security, making such studies essential references in both scientific and policy discussions.

Our findings also highlight the thematic dominance of pollinator decline research within highly cited papers, with 24 of the 40 most‐cited papers focusing on pollinator loss and its ecological consequences. This suggests that research addressing pressing environmental issues tends to attract more citations, particularly when it provides foundational knowledge for conservation strategies. Pollination ecology, as an applied field, may therefore exhibit different citation patterns compared to more theoretical areas of ecology, where methodological and institutional factors might play a larger role. Future research could explore alternative metrics, such as citation velocity (rate of citation accumulation over time) or societal impact indicators, to provide a more nuanced understanding of research influence in pollination ecology.

### 4.4 Trends in Papers and Citations

Trends in publication and citation patterns among pollination ecology researchers reveal key insights into the evolving nature of the field. Our analysis shows a significant increase in pollination ecology publications from 1974, peaking in 2020, followed by a decline. Similarly, total citations grew steadily until 2010 but have since sharply decreased. The fact that citations have declined for over a decade, while publication rates remained stable until 2020, suggests that recent publications are not receiving the same level of recognition as earlier work. This raises important questions about shifts in research focus and the broader impact of newer studies.

One possible explanation for this trend is that, as the field matures, foundational topics such as pollinator importance and global pollinator decline have been extensively covered. Researchers may now be exploring more specialized subfields, including pollinator physiology, taxon‐specific studies, or localized interaction networks. While these specialized studies contribute valuable scientific knowledge, they may not receive as many citations as widely relevant research on pollination services, ecosystem stability, and conservation strategies. Additionally, interdisciplinary shifts may be influencing citation patterns, as pollination ecology research integrates with molecular biology, climate science, or agricultural technology, leading to publications in nontraditional journals that may not be as frequently cited within the core ecological literature.

Another key consideration is the time lag in citation accumulation. Newer studies take time to gain citations, and the observed decline may partly reflect a temporary effect rather than a fundamental reduction in research impact. External factors, such as shifts in funding priorities, changes in journal impact factors, or global disruptions like the COVID‐19 pandemic, may also have played a role in altering publication and citation dynamics. Furthermore, with increasing academic pressures, researchers may be prioritizing the quality of their work over the sheer number of publications, leading to a focus on more rigorous but potentially niche studies. Overall, these findings indicate that the landscape of pollination ecology research is evolving. While a definitive cause of declining citations cannot be determined without further topic‐based analysis, our results suggest that shifts in research focus, interdisciplinary integration, and structural changes in academic publishing may all contribute to these observed trends. Future studies using bibliometric analyses could provide a clearer picture of how pollination ecology research themes have changed over time and whether certain specialized areas are emerging as dominant trends within the field.

### 4.5 Pollination Ecology Is Dominated by Bees

Keyword usage and citation patterns among 223 pollination ecology scientists reveal key trends. Single keywords like “bee,” “plant,” and “pollination” peaked in frequency around 2021 but have since declined while their citations peaked earlier around 2010. Similarly, bi‐gram keywords such as “honey bee” and “ *Apis mellifera* ” followed this pattern with frequency peaking in 2021 but citation declines beginning earlier around 2012 for “ *Apis mellifera* ” and post‐2017 for others. This suggests shifting research focus and citation dynamics over time.

These differences in citation patterns among bi‐gram keywords suggest varying research impacts and evolving scientific interests over time. Our analysis revealed that there have been no significant shifts in research focus or changes in scientific interest within the field of pollination ecology. Topics such as honeybees, particularly *Apis mellifera* , have consistently remained at the forefront of research. Consequently, our findings indicate that pollination ecology has been predominantly centered on research related to bees, with a strong emphasis on honeybees. However, there is a decreasing trend in the number of publications on these research topics approximately after 2020 and a decline in citations after 2010. This trend is consistent with our earlier findings and was anticipated.

## 5 Conclusion

Our analysis of 223 scientists in pollination ecology reveals significant insights into the field's trends and focus areas. Notably, 67.8% of these citations arise from collaborative works, highlighting the importance of interdisciplinary research but also suggesting potential challenges in individual recognition and project management. Analysis of the 40 most‐cited papers showed no significant correlation between citations and factors such as publication year, number of authors, or open‐access status. This suggests that high citation rates are influenced by other factors, including the study's direction and the collaborative nature of the research. Trends in publications and citations show a noticeable increase in pollination ecology research starting around 1974, peaking in 2020, followed by a sharp decline in both publications and citations. This trend suggests a potential shift in research focus or output among pollination ecologists. The decline in citations since 2010 indicates a possible shift toward more specialized or less broadly appealing research topics. Our findings also revealed that pollination ecology has been consistently dominated by research on bees, particularly honey bees ( *Apis mellifera* ). While there has been no significant shift in research focus, the number of publications and citations on these topics has declined since 2020 and 2010, respectively. This decline raises questions about the future direction of pollination ecology research and suggests a need for diversification in research topics to maintain the field's impact and relevance.

One limitation of this study is that our analysis was based on author profiles rather than a traditional bibliometric approach using keyword searches. By selecting scientists known for their work in pollination ecology, we included all their publications, regardless of whether they strictly fell within the domain of pollination ecology. This approach allowed us to assess the overall impact of these researchers but may have overrepresented certain authors' broader body of work while excluding studies from researchers who have contributed to pollination ecology but are not primarily identified as pollination ecologists. A keyword‐based bibliometric approach could complement our method by providing a more systematic and inclusive analysis of research trends in the field.

Additionally, our study focused on publication and citation trends without accounting for the career stages or ages of the researchers analyzed. Since this information is not available in Google Scholar or other public datasets, we were unable to assess how career progression influences research output and citation accumulation. It is possible that the observed peaks and declines in publication and citation trends reflect generational shifts in the scientific community rather than true changes in research focus. Senior scientists may contribute a larger share of highly cited foundational papers, while early‐career researchers may be producing work that has not yet had enough time to accumulate citations. Future research incorporating career‐stage data could provide deeper insights into how individual trajectories shape trends in pollination ecology. Despite these limitations, our analysis offers valuable insights into the evolution of research activity and citation patterns in the field, serving as a foundation for further investigation.

## Author Contributions

**Ehsan Rahimi:** conceptualization (lead), formal analysis (lead), investigation (lead), methodology (lead), writing – original draft (lead). **Chuleui Jung:** project administration (lead), resources (lead), supervision (lead), validation (lead), visualization (lead), writing – review and editing (lead).

## Ethics Statement

The authors have nothing to report.

## Consent

The authors have nothing to report.

## Conflicts of Interest

The authors declare no conflicts of interest.

## Supporting information


Data S1

## Data Availability

Supporting Information Data S1 is available at https://github.com/ehsanrahimi666/Trend‐in‐pollination‐ecology.git.
