# Supplementary material for: Trends in Pollination Scientists' Research: A Comprehensive Analysis in Citations and Research Topics
Source: Ecol Evol. 2025 May 7;15(5):e71215. doi: 10.1002/ece3.71215 (PMC12058208; doi:10.1002/ece3.71215)
Supplement: Supplementary file 1 — Data S1 [file ECE3-15-e71215-s001.zip › Trend-in-pollination-ecology-main/DatS1caption.docx]

The data include a zip file containing figures of all components for authorship analysis, a Word file listing all IDs of the authors, and files of additional figures included in this study.

A zip file listing 223 scholars in pollination ecology along with their papers and citations, named "scholar_publications".

A CSV file listing 250 author names along with their assigned IDs in this study, named "Author_ID".

A CSV file listing 14,661 paper titles along with their authors, journal information, citations, year of publication, and publication and scholar IDs.

A CSV file listing the full information of the 40 most cited papers in this study, named "most_cited_papers".

A CSV file containing and listing 223 author profiles with their related information, named "scholar_profiles".

A CSV file listing the top 20 single keywords with the highest frequency in our dataset's paper titles, named "top_20_words".

A CSV file listing the top 50 bi-gram keywords with the highest frequency in our dataset's paper titles, named " top_50_bigrams_words".
